# Drug Discovery Approaches to Target Wnt Signaling in Cancer Stem Cells

**DOI:** 10.18632/oncotarget.191

**Published:** 2010-10-30

**Authors:** Joshua C. Curtin, Matthew V. Lorenzi

**Affiliations:** ^1^ Oncology Drug Discovery, Research and Development, Bristol-Myers Squibb, Princeton, NJ, USA

**Keywords:** oncotarget, cancer, stem cells, wnt, drug discovery

## Abstract

Cancer stem cells (CSCs) represent a unique subset of cells within a tumor that possess self-renewal capacity and pluripotency, and can drive tumor initiation and maintenance. First identified in hematological malignancies, CSCs are now thought to play an important role in a wide variety of solid tumors such as NSCLC, breast and colorectal cancer. The role of CSCs in driving tumor formation illustrates the dysregulation of differentiation in tumorigenesis. The Wnt, Notch and Hedgehog (HH) pathways are developmental pathways that are commonly activated in many types of cancer. While substantial progress has been made in developing therapeutics targeting Notch and HH, the Wnt pathway has remained an elusive therapeutic target. This review will focus on the clinical relevance of the Wnt pathway in CSCs and tumor cell biology, as well as points of therapeutic intervention and recent advances in targeting Wnt/β-catenin signaling.

## CANCER STEM CELLS: A HIERARCHICAL MODEL

**Figure 1:**
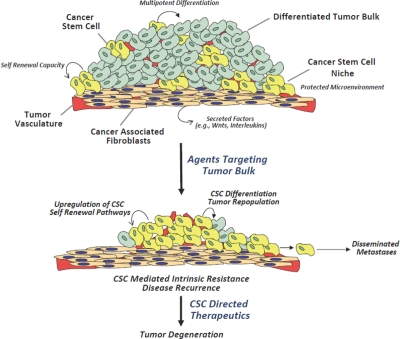
Cancer stem cell properties and therapeutic resistance An illustration of a solid tumor depicts the cellular milieu, comprised of differentiated tumor bulk, a small number of CSCs, and tumor vasculature. The CSC niche provides cues that direct CSCs to undergo self-renewal to maintain the CSC sub-population, or differentiation to generate the tumor bulk. Conventional chemotherapeutic approaches target the tumor bulk, but due to their inherent chemoresistance, CSCs remain largely unaffected and potentially lead to tumor repopulation and/or metastasis. CSC directed therapeutics that target critical regulatory pathways in CSCs, such as Wnt, Notch and HH, have the potential to inhibit tumor repopulation and metastasis, resulting in tumor degeneration.

Cancer stem cells (CSCs) represent the apex in the hierarchical model of tumor genesis, heterogeneity and metastasis [1-4]. Analogous to normal stem cells, CSCs are thought to possess the capacity for unlimited self-renewal through symmetric cell division, the ability to give rise to progeny cells through asymmetric division, and also an innate resistance to cytotoxic therapeutics (Figure 1) [5,6]. While the process of differentiation initiated by a normal stem cell ultimately results in a specialized progeny with no proliferative potential, a CSC gives rise to progeny that do not undergo terminal differentiation but instead exhibit uncontrolled proliferation. In the case of solid tumors, this process drives formation of the bulk tumor mass. This model is in contrast to the clonal evolution model, which proposes that tumors arise from a precursor cell with a competitive growth advantage, most likely due to the accumulation of mutations that allow unchecked cell division and evasion of the apoptotic process [7]. The clonal evolution model would predict that all cells within a given tumor are phenotypically homogeneous; indeed, cytotoxic agents that indiscriminately target proliferating cells constitute the majority of anti-cancer agents in the clinic. In some cases, these agents are initially very effective at reducing or eliminating tumor burden in the patient. However, tumors often recur, develop resistance and metastasize. Furthermore, heterogeneity is a hallmark of tumors in the clinic.

Due to their similarities to normal stem cells, CSCs are predicted to rely on pathways that govern development, self-renewal and cell fate. In embryonic stem cells, these processes are in large part regulated by three signaling programs: the Wnt, Notch and Hedgehog (HH) pathways [8-10]. It is an intriguing finding, therefore, that these pathways are frequently dysregulated in many types of cancers, and specifically within subpopulations of these cancers that possess stem-like properties [2,11-13]. From a drug development perspective, this provides an opportunity not only for new classes of targeted agents, but also a novel targeting paradigm: the prospect of targeting cells responsible for tumor initiation, progression, and even metastasis. Furthermore, as CSCs often display an inherent resistance to many standard cytotoxic agents [14-17], targeting CSCs is also an attractive strategy for overcoming drug resistance. Agents targeting the Notch and HH pathways have shown pre-clinical promise, and are currently being evaluated in clinical trials [18,19]. While the Wnt pathway has been more challenging to target, several recent advances have been made with regard not only to new therapeutic agents, but new targets within the pathway, as well. This article focuses on the significance of Wnt signaling in tumor cell and CSC biology, and strategies for therapeutic intervention within Wnt pathway.

## DEVELOPMENTAL PATHWAYS AS THERAPEUTIC TARGETS

Since the developmental pathways important to normal stem cells are also important to CSCs, a great deal of time has gone into developing therapeutic agents targeting these pathways. The main focus of this review is the Wnt/β-catenin pathway. However, due to the similarities in signaling components and crosstalk among these pathways, it is important to provide a brief review of the Notch and HH signaling cascades, where progress has outpaced efforts in the Wnt pathway. These pathways share overarching themes such as myriad permutations of ligand/receptor interactions that ultimately impinge and rely heavily upon a central molecule in the signal transduction cascade: lessons learned from approaches successful in targeting Notch and HH pathways may provide valuable insight in how to approach the Wnt signaling pathway.

## NOTCH

Notch signaling regulates numerous processes in both embryonic development and in adult tissue renewal [20]. During embryogenesis, Notch is critical in neuronal and pancreatic development. In the adult organism, Notch regulates the fate of hematopoietic stem cells and gastrointestinal stem cells, as well playing a role in angiogenesis [21,22]. Aberrant Notch signaling has been observed in hematopoietic tumors, such as T-ALL, and solid tumors, such as non-small cell lung carcinoma (NCSCL), breast cancer, and various brain cancers [23-28]. The Notch signaling pathway is comprised of four membrane-bound receptors (Notch 1-4) and five membrane-tethered ligands (DLL 1, 3, 4, and Jagged 1, 2) [20,29]. A complex signaling cascade is initiated when a ligand expressed on one cell engages a receptor expressed on the surface of another cell, and is thus dependent on cell-cell interactions. Upon ligand/receptor interaction, a cleavage event removes the Notch/ligand complex from the membrane-bound portion of Notch. The cytoplasmic region of Notch then undergoes a proteolytic cleavage mediated by γ-secretase, releasing an intracellular domain peptide that translocates to the nucleus and drives transcription of Notch target genes. These target genes, including HES family members and myc, regulate diverse cellular processes such as tissue renewal and proliferation.

The numerous steps required to translate ligand binding to target gene activation provide a series of potential points of therapeutic intervention in the Notch signaling pathway. As a result, there are currently numerous preclinical therapeutics under evaluation, as well as several clinical trials involving Notch pathway inhibitors. Preclinical agents including monoclonal antibodies (mAbs) targeted against Notch and Notch ligands are meant to disrupt ligand/receptor interaction events [30,31]. While this approach has shown promise, the large number of receptor/ligand permutations may ultimately result in limited efficacy. γ-secretase inhibitors act downstream of ligand/receptor interactions and therefore should not be affected by the diversity of ligands, receptors, and possible combinations thereof. Several γ-secretase inhibitors are currently being evaluated in Phase I and II clinical trials [18,32-35].

## HEDGEHOG

Under normal conditions, HH signaling plays important roles in embryonic development and is also involved in tissue regeneration in adults [36,37]. Activating events in the HH pathway are involved in numerous human cancers, including pancreatic cancer, various leukemias, and basal cell carcinoma (BCC) [38-45]. Like Notch signaling, HH signaling is comprised of multiple ligands that can regulate receptor activity [36,37]. Mammalian HH signaling is initiated when one of three HH ligands- Sonic, Indian, and Desert HH- bind the dodecatransmembrane receptor Patched (Ptch1). Ligand/receptor interactions occur through an autocrine or paracrine manner, depending on the context. Receptor engagement results in activation of the heptatransmembrane Smoothened (Smo), which is held in an inactive state in the absence of ligand. Smo activation in turn regulates the activity of transcription factors Gli1, Gli2 and Gli3. Gli1/2/3 function to regulate transcription of genes involved in HH signaling such as Gli1 and Ptch1, and importantly genes involved in epithelial-mesenchymal transition (EMT), such as SNAIL1 [36,37].

Dysregulation of nearly every step of the HH signaling pathway has been linked to cancer progression. For example, mutations in Ptch1, Smo, Gli1 and Gli3 are linked to BCC, medullablastoma and pancreatic cancer [40,42,43,46-48]. Ligand upregulation can also drive cancer formation, as has been described in breast, ovarian, pancreatic, prostate and lung cancers [41,49-52]. Not only does this list illustrate the relevance of HH signaling in human cancers, it also provides multiple candidates to target with novel agents. In fact, several agents targeting the HH pathway have shown encouraging pre-clinical results, and are currently in Phase I and Phase II clinical trials [19,48,53,54]. To date, these trials are evaluating the effects Smo antagonists developed by multiple pharmaceutical companies in a broad spectrum of cancers, such as BCC, multiple myeloma, brain, breast and gastric cancers. Smo is an attractive target, due to its centrality in the HH pathway. It will be of interest to see if agents targeting other components of HH signaling achieve pre-clinical validation. To this end, efforts are currently underway to target the Gli transcription factors with small molecules [55,56], as well antibody neutralization of HH ligands to block signaling at the ligand/receptor interface [57]. While current clinical trials are assessing HH inhibition in frank disease, the role of HH in CSCs and EMT suggests that this type of therapy may also be beneficial in combination with other chemotherapies, such as traditional cytotoxic agents, to prevent re-initiation of tumorigenesis and/or metastasis by CSCs.

## WNT

Elucidation of the Wnt signaling pathway was built upon the seminal observation that the Drosophila segment polarity gene Wingless had a common origin with the murine oncogene Int-1 [58]. This discovery launched an intense field of study that has resulted in the identification of 19 mammalian Wnts, myriad Wnt receptors, and characterization of pathways involved in biological processes involved in embryogenesis, development, cell polarization, differentiation and proliferation [59-62].

Wnts are secreted glycoproteins which bind to cell surface receptors to initiate signaling cascades important in many physiological settings, as described above [63]. Wnt signaling cascades are highly conserved among species, and have traditionally fallen into two categories: canonical and non-canonical, differentiated by their dependence on β-catenin. Non-canonical Wnt pathways, such as the planar cell polarity (PCP) and Ca^2+^ pathway, regulate processes such as cell dorsoventral patterning and neuronal migration, function through β-catenin independent mechanisms, and remain better characterized in lower organisms [64-66]. Canonical Wnt signaling is initiated when a Wnt ligand engages co-receptors of the Frizzled (Fzd) and low-density lipoprotein receptor-related protein (LRP) families, ultimately leading to β-catenin stabilization, nuclear translocation and activation of target genes [67-72]. This canonical pathway is far better characterized in mammalian systems, and will be the focus of this review. We will highlight paradigms and discovery efforts that highlight the promise, and challenges, of targeting the Wnt/β-catenin pathway; for a more exhaustive review of discovery, pre-clinical and clinical candidates, we recommend several excellent review articles found among the references [11,73-75].

**Figure 2:**
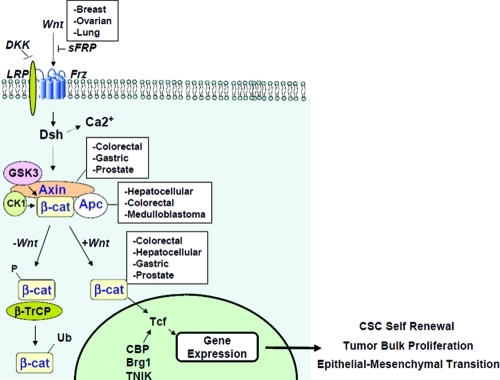
Canonical Wnt signaling and dysregulation in cancer The Wnt signaling pathway is comprised of extracellular, cytoplasmic and nuclear signaling events that are amenable to therapeutic intervention. Dysregulation at these stages are common in numerous cancers, captured in the white boxes. Upon entering the nucleus and interacting with TCF/LEF and various co-activators, β-catenin drives transcription of programs critical for CSCs, tumor cells and EMT.

It has been appreciated for decades that dysregulation of the mechanisms that regulate β-catenin signaling is a common feature across a broad spectrum of human cancers (Figure 2). However, as β-catenin is an intracellular signaling protein with no discernable enzymatic activities, it has been considered to be “undruggable”. Due to intense research efforts in this field, recent findings have provided hope that we may be able to target other aspects of this pathway to inhibit aberrant β-catenin activation/transcriptional activity.

In the absence of Wnt stimulus, β-catenin is held in an inactive state by a multimeric “destruction” complex comprised of adenomatous polyposis coli (APC), Axin, glycogen synthase kinase 3β (GSK3β) and casein kinase 1α (CK1α) [76]. APC and Axin function as a scaffold, permitting GSK3β- and CK1α-mediated phosphorylation of critical residues within β-catenin. These phosphorylation events mark β-catenin for ubiquitination and subsequent proteasomal degradation [77,78].

An additional layer of regulation that keeps β-catenin levels low in cells is the expression and secretion of antagonists of the Wnt pathway. These come in two flavors, proteins that bind Wnt ligands, and proteins that bind Wnt receptors [79]. Members of the secreted Frizzled-related protein (sFRP) family, as well as Wnt Inhibitory Factor-1 (WIF-1) and Cerberus function in a manner analogous to decoy receptors by binding Wnts and preventing their interaction with Fzd/LRP [80-85]. A second family of secreted Wnt antagonists is the Dickkopf (Dkk) family [86]. Dkk proteins bind to LRP5/6, thus preventing formation of the Wnt-Fzd-LRP5/6 ternary complex required to initiate canonical Wnt signaling [87-89].

Canonical Wnt/β-catenin signaling is initiated when a Wnt ligand engages its co-receptors, Fzd and LRP5/6, forming a ternary complex on the extracellular membrane. Fzd, made up of 10 family members, is a seven transmembrane receptor that binds Wnts through an extracellular cysteine-rich domain (CRD) [90]. LRP5/6 is a transmembrane receptor with a large extracellular domain critical for Wnt binding, and a short intracellular tail that plays an important role in initiating the Wnt-mediated signaling cascade [91-93]. These receptors will be described in greater detail below, as they play critical roles in initiating Wnt signaling and are thus attractive points for therapeutic intervention.

Wnt binding to Fzd-LRP5/6 initiates a cascade of events that results in disruption of the β-catenin destruction complex, leading to β-catenin stabilization and nuclear translocation. The early events initiated by Wnt-Dvl-LRP binding remain incompletely understood. However, current data demonstrate that Wnt binding results in phosphorylation of the cytoplasmic tail of LRP5/6 within a PPSP domain in its C-terminus [87]. This phosphorylation involves GSK3β and casein kinase 1-gamma (CK1γ), though it is unknown if other kinases or phosphatases regulate LRP5/6 phosphorylation. Following phosphorylation, Axin and GSK3β are recruited to LRP5/6, thereby driving dissociation of the destruction complex. Concurrent with Axin recruitment is recruitment and phosphorylation-mediated activation of the Dishevelled (Dvl) family of proteins. CK1 family members play a role in Dvl activation [94], and Dvl has also been implicated in the phosphorylation of LRP5/6 [95,96]. While the precise series of events remains to be clarified, it is generally accepted that Wnt binding to Fzd and LRP5/6 results in recruitment of Axin, GSK3β and Dvl to the co-receptor complex, leading to disruption of the β-catenin destruction complex, β-catenin stabilization and nuclear translocation. Once in the nucleus, β-catenin forms a complex with members of the T-cell factor/lymphoid enhancer factor (TCF/LEF) family of transcription factors, recruiting co-factors such as CBP, p300, TNIK, Bcl9 and Pygopus, and ultimately driving transcription of target genes including c-myc, cyclin D, and survivin [95-97].

## WNT SIGNALING IN CANCER AND CANCER STEM CELLS

The relevance of Wnt signaling in human cancers is highlighted by the frequency with which this pathway is aberrantly activated across a vast range of malignancies. The first described, and perhaps best well known role for Wnt/β-catenin signaling is in colon cancer, where nearly 90% of these tumors harbor mutations that result in β-catenin mutation [98-100]. The most common type of mutation in colon cancer results in the inactivation of APC, thus driving constitutive activation of β-catenin. Activating mutations within β-catenin itself are also found in this disease, albeit at a much lower frequency [101,102]. It is important to note that while there are numerous mechanisms that drive aberrant Wnt/β-catenin signaling, these different mechanisms nearly always occur in a mutually exclusive manner. It is very rare, for example, to find a colorectal tumor with mutations in both APC and β-catenin.

Interestingly, a growing body of evidence illustrates a critical role of β-catenin in CSCs [103-105]. For example, stem-like colon cells with a high level of β-catenin signaling have a much greater tumorigenic potential than counterpart cells with low β-catenin signaling [106]. Furthermore, hematopoietic stem cell (HSC) function is regulated by Wnt activity [107-109]. HSCs and the niche microenvironments in which they reside secrete Wnts, again illustrating a possible autocrine or paracrine Wnt model [108]. Axin expression in HSCs leads to growth inhibition and diminished reconstitution, and HSC function and lymphocyte development have been shown to be dependent on Wnt signaling [109].

## STRATEGIES FOR TARGETING THE WNT PATHWAY

The brief overview of canonical Wnt signaling described above provides a glimpse into the complexity of this system. However, the array of numerous ligands, receptors, kinases, signal transducers and transcription factor complexes also provide an opportunity for multiple modes of therapeutic targeting and intervention. We will therefore discuss three major areas of targeting the Wnt pathway which have shown promise in recent years: receptor/ligand interactions, cytosolic signaling components, and nuclear signaling components.

## RECEPTOR/LIGAND INTERACTIONS

Mutations downstream of Wnt receptors, such as those found in APC or β-catenin, were the first examples of aberrant Wnt signaling in human cancers. Some cancers, however, demonstrate hallmarks of constitutive Wnt signaling in the absence of downstream mutations. Triple negative breast cancers and non-small cell lung cancers (NSCLCs) have been demonstrated to harbor high levels of uncomplexed cytosolic β-catenin and exhibit a high basal level of Wnt/β-catenin transcriptional activation [110,111]. This suggests an autocrine mechanism of Wnt activation in certain tumors. Indeed, epigenetic silencing of endogenous Wnt inhibitors such as sFRP has been observed in many types of cancers [112,113]. Furthermore, certain tumor cell lines have been shown to express high levels of particular Wnts, and treatment of these cells with Wnt inhibitors such as Dkk or sFRP has an anti-proliferative effect [110,111]. In line with these findings are other observations and opportunities that validate the approach of targeting Wnt signaling at the extracellular level.

Given the sheer number of Wnt growth factors, and functional redundancy demonstrated by knock-out mouse models, it is tempting to speculate that antibodies directed against any particular Wnt may not be a viable approach for inhibiting the Wnt pathway. However, a series of studies by the Jablons group has demonstrated that certain tumor models rely heavily on specific Wnts. Monoclonal antibodies against Wnt1 and Wnt2 drive apoptosis in a variety of tumor models, including melanoma, NSCLC, mesothelioma, sarcoma, breast and CRC cells [97,114-118]. However, these antibodies only had modest efficacy in xenograft models derived from some of these cells [115,118]. This could potentially be explained by differences in Wnt expression profiles in mice relative to a specific tissue culture line. Murine and human Wnts, as well as their receptors, share a high degree of homology, and have been demonstrated to function interchangeably. Nevertheless, these *in vitro* results are encouraging, and warrant further exploration into the development of specific Wnt antibodies.

While tumors which rely exclusively on a specific Wnt may be amenable to monoclonal antibody targeting, tumors that are driven by multiple Wnt ligands would not be effectively targeted in this manner. In this scenario, a pan-Wnt inhibitor may prove to be more efficacious. A recent study from Genentech demonstrated that a soluble ligand binding domain of Fzd8, Fzd8-CRD-Fc, inhibited autocrine Wnt signaling *in vitro*, as well as in multiple xenograft models [119]. Mice treated with this soluble receptor displayed no signs toxicity after several weeks of treatment, demonstrating that pan-Wnt inhibition may be a safe and efficacious approach for targeting appropriate tumor types.

**Table 1:**
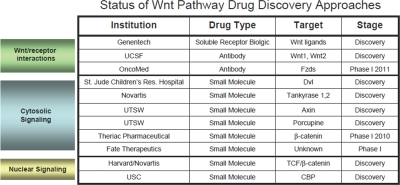
Wnt pathway drug discovery approaches A summary of various Wnt therapeutics in early development or Phase I

An alternative approach to inhibiting ligand/receptor interactions would be to target the Wnt co-receptors, LRP5/6 and members of the Fzd family. The LRP family of co-receptors is comprised of 2 highly homologous members, LRP5 and LRP6. These are long single-pass transmembrane receptors, and endogenous Wnt inhibitors of the Dkk family bind LRP5/6 to block Wnt signaling [86,120]. Thus, a plausible approach to Wnt inhibition may be through antibody targeting of LRP5/6. Furthermore, a recent study by Bourhis et al. described specific domains with the propeller regions of LRP6 that may preferentially bind specific Wnt ligands [121]. These propeller domains may offer an added degree of specificity for antibody-mediated Wnt inhibition.

The Fzds are family of seven transmembrane receptors comprised of 10 members. Fzd family members share a high degree of homology in their extracellular cysteine-rich domain (CRD), which is the region responsible for Wnt-Fzd interactions [90]. While not thought to function in as classical G-protein coupled receptors, the transmembrane regions of Fzds may still serve as potential small molecule binding pockets, raising the possibility that this approach may be a viable approach. An antibody that recognizes the conserved CRD regions across multiple Fzds may also prove to be an attractive therapeutic strategy for blocking Wnt signaling at the receptor level.

## CYTOSOLIC SIGNALING COMPONENTS

Biologics approaches interfering with Wnt/co-receptor interactions have shown promise, as illustrated above. The intracellular signaling components of the Wnt pathway also provide myriad targets for therapeutic intervention, and are ripe for targeting with either small molecules or perhaps protein mimetics designed to impact critical steps along the Wnt signaling cascade.

Constitutive β-catenin signaling, due to inactivating mutations in APC or activating mutations within β-catenin itself, plays a critical role in the development of certain cancers. Colorectal cancer is the best example, as nearly 90% of all CRCs harbor mutations driving β-catenin signaling. These mutations lead to the stabilization and accumulation of β-catenin and subsequent translocation into the nucleus. Preventing β-catenin stabilization and accumulation are of obvious interest, though this has proven to be an extremely difficult task, perhaps because β-catenin does not possess enzymatic activity. However, a greater understanding of the β-catenin destruction complex and the auxiliary proteins involved in its regulation provide new possibilities for therapeutic intervention to regulate this step of the Wnt signaling pathway.

The most upstream intracellular components of the Wnt pathway are at the receptor level. LRP5/6 phosphorylation plays a critical role in initiating the Wnt signaling cascade [122]. While the series of events leading to this phosphorylation are not yet entirely clear, this is an area of intense research and will most likely yield new clues, and thus targets, regarding intracellular LRP5/6 phosphorylation. Similarly, while Dvl family proteins play a critical role in disruption of the β-catenin destruction complex, the precise mechanism of their regulation remains to be elucidated. Several compounds were recently described that target the PDZ domain of Dvl and subsequently interfere with Wnt/β-catenin signaling [123,124]. CK1 family members play a positive role in phosphorylation and activation of Dvl, phosphorylation of LRP5/6 and phosphorylation of β-catenin [87,94,125], and may therefore provide an opportunity for intervention. However, different isoforms within the CK1 family play opposing roles in regulating Wnt signaling: CK1δ contributes to LRP phosphorylation and CK1ε phosphorylates Dvl, while CK1α phosphorylates β-catenin, thus playing a negative regulatory role in the pathway. A CK1 modulator must therefore demonstrate the appropriate selectivity. Similarly, GSK3β also plays a critical role within the destruction complex, and molecules which potentiate GSK3β activity in the setting of hyperactive Wnt signaling provide a therapeutic benefit. With the exception of LRP5/6, it will be important to keep in mind that many of these kinases play important roles in signaling programs distinct from the Wnt pathway, and will therefore need to be approached in a highly context-dependent manner.

A critical component of the β-catenin destruction complex is the scaffolding protein Axin. Axin overexpression can inhibit Wnt signaling, and Axin undergoes genetic inactivation in various cancers [126-128]. This suggests that positive regulation of Axin activity may have a negative impact on Wnt signaling. Interestingly, a recent study utilizing a high throughput screen to identify inhibitors of Wnt-mediated transcriptional activity identified XAV939, a small molecule that increased levels of Axin1 and Axin2, thus inhibiting β-catenin stabilization, accumulation and transcriptional activity [129]. The authors then went on to show that XAV939 inhibited the activity of Tankyrase 1 and Tankyrase 2, members of the PARP family that mark Axin for degradation [130]. In a similar set of studies, Chen et al. independently identified IWR-1, a small molecule that increased Axin stabilization [131]. Both compounds inhibited Wnt signaling and tailfin generation in zebrafish, a classical Wnt/β-catenin driven model. IWR-1 inhibited β-catenin activity in DLD-1 cells, a human CRC cell line that harbors an inactivating mutation in APC, and XAV939 inhibited growth in the same setting. These data offer encouragement that in the appropriate setting, Axin stabilization may provide a novel strategy to inhibit β-catenin signaling. However, the effect of these compounds in the setting of an activating mutation in β-catenin remains to determined. Furthermore, the effects of systemic Axin stabilization and Tankyrase inhibition have not yet been investigated. Compounds such as XAV939 and IWR-1 will surely help us gain a greater understanding these aspects of Tankyrase and Axin biology, and may guide efforts of identifying cancer types susceptible to this mechanism of action.

Ultimately, these myriad upstream signaling events impinge upon β-catenin, leading to its stabilization, accumulation and nuclear translocation. Despite intense research efforts, progress has been slow in directly and selectively targeting β-catenin. Theriac Pharmaceutical Corporation recently described a small molecule inhibitor of the Wnt pathway which is proposed to function via inducing β-catenin destabilization [73]. CWP232291 was identified in a high throughput screen for inhibitors of Wnt/β-catenin mediated transcriptional activity. *in vitro*, CWP232291 demonstrated anti-proliferative effects in various cell lines, and inhibited transcription of β-catenin target genes. In an *in vivo* AML model, CWP232291 inhibited tumor progression and exhibited a favorable safety profile, and is currently scheduled for Phase I clinical trials in AML and multiple myeloma in 2010. While its mechanism of action remains to be elucidated, this compound was reported to be active in the context of both wild-type and mutant β-catenin, raising the possibility of anti-tumor effects across a broad range of cancers.

Several agents with diverse, or even unknown, mechanisms of action have shown activity in Wnt/β-catenin driven cancers and cancer cell lines. Aspirin and non-steroidal anti-inflammatory drugs (NSAIDs) have recently shown promise in clinical trials at preventing polyp formation in colon cancer patients without mutations in APC, and regular use of these agents has been correlated with a decreased occurrence of cancers such as breast and lung cancer [132-134]. While these are non-specific agents that regulate numerous cellular processes, cell culture experiments have demonstrated that various NSAIDs can inhibit β-catenin nuclear localization and Wnt/β-catenin mediated gene transcription [135]. Other compounds, such as the polyphenols curcumin and ECGC, also inhibit Wnt/β-catenin activity in cellular assays, though their mechanism(s) of action remain undefined [136,137].

An additional component of Wnt signaling, especially important in those tumors in which paracrine/autocrine Wnt signaling drives activation of the pathway, is the processing and secretion of the Wnt ligands. In the same study mentioned above that led to the discovery of the Axin destabilizer IWR-1, IWP-1 and IWP-2 were shown to inhibit Wnt-driven transactivation activity with similar potency, though through a distinct mechanism [131]. IWPs were shown to down-regulate Wnt secretion by inhibiting the activity of the acyltransferase Porcupine (Porcn). Porcn belongs to the family of membrane-bound O-acyltransferases (MBOATs), which facilitate protein secretion via palmitoylation [138-141]. IWPs inhibited the secretion of Wnts, but not other MBOAT substrates, indicating a degree of Wnt specificity. While its precise mechanism of Porcn inhibition is unclear, this paradigm demonstrates another layer of Wnt signaling that may be amenable to pharmacological inhibition.

## NUCLEAR SIGNALING COMPONENTS

Upon entering the nucleus, β-catenin interacts with members of the TCF/LEF family of transcription factors to drive target gene expression. In the absence of β-catenin, TCF/LEF is held in a transcriptionally inactive state through interactions with co-repressors such as Groucho and HDACs [142]. β-catenin interaction with TCF/LEF displaces these co-repressors and recruits a variety of co-activators, such as CBP, p300, BCL9, Pygopus, and Brg1 [142-144]. These co-activators play critical roles in driving β-catenin-mediated transcription, and therefore represent potential therapeutic targets.

A compelling series of studies by the Kahn group has suggested differential roles for the highly homologous CBP and p300 in Wnt/β-catenin-driven signaling, especially with regard to the role of the Wnt pathway in CSCs [75,145-147]. A screen of compounds that could inhibit β-catenin/TCF-dependent transactivation identified ICG-001, which also down-regulated β-catenin target genes and inhibited growth in a CRC xenograft model [146]. ICG-001 was shown to disrupt selectively the interaction between β-catenin/TCF and CBP, but not p300. While these data are encouraging, it is important to note that ICG-001 targets CBP, a promiscuous co-factor involved in numerous signaling pathways. Furthermore, as the effective dose of ICG-001 is near 10μM, it will be critical to develop derivatives with improved specificity in order to rule out off-target effects. These studies offer hope that targeting distinct β-catenin/TCF co-factor interactions may provide an opportunity to target specific sub-populations of Wnt-dependent cells.

Leproucelet and colleagues utilized a high throughput screen to identify a series of small molecules that disrupted β-catenin/TCF interaction [148]. These compounds inhibited β-catenin/TCF transactivation activity and target gene activity in HCT116 CRC cells, and inhibited duplication of the Xenopus embryonic dorsal axis. The mechanism(s) by which these compounds disrupt β-catenin/TCF interaction remain to be elucidated, but provide a proof of concept that interference of this protein-protein interaction can be achieved with promising effects.

An expanded base of knowledge regarding critical β-catenin/TCF co-regulators will be critical in the development of novel antagonists of the Wnt signaling pathway. For example, recent reports from the Clevers and Yamada groups have identified Traf2- and Nck-Interacting Kinase (TNIK) as a critical mediator of Wnt-driven transcription in CRC [149,150]. In the mouse small intestinal crypt, TNIK interacts with the β-catenin/TCF complex exclusively in the proliferative crypt, as opposed to the differentiated villus, in a β-catenin dependent manner. TNIK phosphorylates TCF4, and a kinase-dead mutant of TNIK abrogated β-catenin/TCF-driven transactivation. siRNA-mediated inhibition of TNIK had an anti-proliferative effect *in vitro*, and inhibited tumor growth in an HCT116 xenograft model. It will be of interest to determine if inhibitors of this component of the β-catenin/TCF transcriptional complex, as well as the others listed above, serve as novel targets of inhibition in the Wnt signaling pathway.

## MOVING FORWARD: TARGETING WNT IN CANCER AND CANCER STEM CELLS

As a result of its role in numerous cancers, the Wnt signaling pathway is a prime target for therapeutic intervention. Inhibition of Wnt signaling has proven to be an elusive goal over the years. However, as new developments emerge in the field of Wnt biology, the field continues to inch closer to an effective strategy of Wnt inhibition. Concurrent with these advances comes the realization that Wnt signaling plays critical roles in biology of CSCs.

A role for Wnt signaling has been demonstrated in a variety of CSC settings, including colon, breast and cutaneous CSCs, as well as in hematopoietic stem cells [104-106,151-153]. CSCs are also thought to play a role in drug resistance and metastasis [16,154-158]. The approaches described above for targeting various components of the Wnt signaling pathway may potentially also be used to target CSCs. For example, colon cancer stem cells have been described to harbor high levels of β-catenin transcriptional activity, and this is correlated with their tumorigenic potential [106]. Wnt stimulation may occur in an autocrine fashion due to colon CSC Wnt secretion, and may be potentiated by secreted factors from cells within the CSC microenvironment. It is therefore plausible to imagine a therapeutic strategy involving several of the potential agents listed above: a Wnt neutralizing approach or Wnt secretion inhibitor may dampen initiation of the Wnt signaling cascade, while a β-catenin destabilizer or β-catenin/TCF disruptor could provide downstream inhibition. Wnt inhibition could also be used in combination with classic chemotherapeutic agents. A cytotoxic agent such as cisplatin may target the bulk of a tumor but not the inherently chemoresistant CSCs, which might ultimately give rise to chemorefractory tumor cells. However, if the CSCs were targeted in parallel with a Wnt pathway inhibitor, a curative response might be achieved. These situations are clearly speculative, but are meant to highlight the enormous potential of targeting developmental pathways in cancer and CSCs, such as the Wnt signaling pathway.

Another potential advantage of employing a Wnt-targeted therapy is the potential role for CSCs in resistance to classical cytotoxic treatments (i.e. chemotherapy and ionizing radiation) and in metastatic disease. CSCs in a broad range of cancers are relatively more resistant to these conventional therapeutic approaches than their bulk tumor cell counterparts, as has been described in CSCs in leukemias and breast, colorectal, and brain cancers [16,17,159,160]. A role for autocrine Wnt signaling has been described in various breast cancer cell lines: certain triple-negative breast cancer lines have been shown to express Wnt ligands, and harbor hallmarks of aberrant Wnt/β-catenin signaling in the absence of common mutations in the pathway [111]. Wnt signaling in these cells is inhibited by overexpression of endogenous inhibitors such as Dkk1, thus validating an autocrine Wnt loop that is amenable to pharmacologic inhibition. In breast cancer patients, a recent study has also demonstrated the presence of CD44^+^CD24^low^ stem-like cells in metastases, suggesting a role for breast CSCs in the metastatic process of this disease [154,161]. Furthermore, the Wnt pathway regulates epithelial-mesenchymal transition (EMT), an important component of metastasis [162-165]. During development, Wnt signaling plays a critical role in EMT required for heart cushion development, and aberrant Wnt signaling also drives EMT and tumor formation in mouse xenograft models [162-165]. Cells undergoing EMT possess important properties normally found in stem cells, including the acquisition of the CD44^+^CD24^low^ cell-surface marker pattern and the ability to form spheroids in suspension culture- key properties of normal and cancer stem cells [165,166]. It is therefore reasonable to hypothesize that the Wnt pathway may offer a unique opportunity to target metastasis, which is the leading cause of morbidity in many types of cancers.

## References

[1] Jordan CT, Guzman ML, Noble M (2006). Cancer stem cells. N Engl J Med.

[2] Li L, Clevers H (2010). Coexistence of quiescent and active adult stem cells in
mammals. Science.

[3] Snippert HJ, Haegebarth A, Kasper M, Jaks V, van Es JH, Barker N, van de Wetering M, van den Born M, Begthel H, Vries RG, Stange DE, Toftgard R, Clevers H (2010). Lgr6 marks stem cells in the hair follicle that generate all cell
lineages of the skin. Science.

[4] Weissman IL (2000). Stem cells: units of development, units of regeneration, and units in
evolution. Cell.

[5] Reya T, Morrison SJ, Clarke MF, Weissman IL (2001). Stem cells, cancer, and cancer stem cells. Nature.

[6] Visvader JE, Lindeman GJ (2008). Cancer stem cells in solid tumours: accumulating evidence and
unresolved questions. Nat Rev Cancer.

[7] Shackleton M, Quintana E, Fearon ER, Morrison SJ (2009). Heterogeneity in cancer: cancer stem cells versus clonal
evolution. Cell.

[8] Dickinson ME, McMahon AP (1992). The role of Wnt genes in vertebrate development. Curr Opin Genet Dev.

[9] Kintner C (1992). Molecular bases of early neural development in Xenopus
embryos. Annu Rev Neurosci.

[10] Ruiz i Altaba A (1999). Gli proteins and Hedgehog signaling: development and
cancer. Trends Genet.

[11] Barker N, Clevers H (2006). Mining the Wnt pathway for cancer therapeutics. Nat Rev Drug Discov.

[12] Haegebarth A, Clevers H (2009). Wnt signaling, lgr5, and stem cells in the intestine and
skin. Am J Pathol.

[13] Wang Y, Krivtsov AV, Sinha AU, North TE, Goessling W, Feng Z, Zon LI, Armstrong SA (2010). The Wnt/beta-catenin pathway is required for the development of
leukemia stem cells in AML. Science.

[14] Costello RT, Mallet F, Gaugler B, Sainty D, Arnoulet C, Gastaut JA, Olive D (2000). Human acute myeloid leukemia CD34+/CD38- progenitor cells have
decreased sensitivity to chemotherapy and Fas-induced apoptosis, reduced
immunogenicity, and impaired dendritic cell transformation
capacities. Cancer Res.

[15] de Grouw EP, Raaijmakers MH, Boezeman JB, van der Reijden BA, van de Locht LT, de Witte TJ, Jansen JH, Raymakers RA (2006). Preferential expression of a high number of ATP binding cassette
transporters in both normal and leukemic CD34+CD38- cells. Leukemia.

[16] Eramo A, Ricci-Vitiani L, Zeuner A, Pallini R, Lotti F, Sette G, Pilozzi E, Larocca LM, Peschle C, De Maria R (2006). Chemotherapy resistance of glioblastoma stem cells. Cell Death Differ.

[17] Ishikawa F, Yoshida S, Saito Y, Hijikata A, Kitamura H, Tanaka S, Nakamura R, Tanaka T, Tomiyama H, Saito N, Fukata M, Miyamoto T, Lyons B, Ohshima K, Uchida N, Taniguchi S (2007). Chemotherapy-resistant human AML stem cells home to and engraft within
the bone-marrow endosteal region. Nat Biotechnol.

[18] Luistro L, He W, Smith M, Packman K, Vilenchik M, Carvajal D, Roberts J, Cai J, Berkofsky-Fessler W, Hilton H, Linn M, Flohr A, Jakob-Rotne R, Jacobsen H, Glenn K, Heimbrook D (2009). Preclinical profile of a potent gamma-secretase inhibitor targeting
notch signaling with in vivo efficacy and pharmacodynamic
properties. Cancer Res.

[19] Robarge KD, Brunton SA, Castanedo GM, Cui Y, Dina MS, Goldsmith R, Gould SE, Guichert O, Gunzner JL, Halladay J, Jia W, Khojasteh C, Koehler MF, Kotkow K, La H, Lalonde RL (2009). GDC-0449-a potent inhibitor of the hedgehog pathway. Bioorg Med Chem Lett.

[20] Fortini ME (2009). Notch signaling: the core pathway and its posttranslational
regulation. Dev Cell.

[21] Brabletz S, Schmalhofer O, Brabletz T (2009). Gastrointestinal stem cells in development and cancer. J Pathol.

[22] Zhou K, Huang L, Zhou Z, Hu C, Liu W, Zhou J, Sun H (2010). Wnt and Notch signaling pathways selectively regulating
hematopoiesis. Ann Hematol.

[23] Allenspach EJ, Maillard I, Aster JC, Pear WS (2002). Notch signaling in cancer. Cancer Biol Ther.

[24] Berman JN, Look AT (2007). Targeting transcription factors in acute leukemia in
children. Curr Drug Targets.

[25] Guan E, Wang J, Laborda J, Norcross M, Baeuerle PA, Hoffman T (1996). T cell leukemia-associated human Notch/translocation-associated Notch
homologue has I kappa B-like activity and physically interacts with nuclear
factor-kappa B proteins in T cells. J Exp Med.

[26] Roy M, Pear WS, Aster JC (2007). The multifaceted role of Notch in cancer. Curr Opin Genet Dev.

[27] Shih Ie M, Wang TL (2007). Notch signaling, gamma-secretase inhibitors, and cancer
therapy. Cancer Res.

[28] Pannuti A, Foreman K, Rizzo P, Osipo C, Golde T, Osborne B, Miele L (2010). Targeting Notch to target cancer stem cells. Clin Cancer Res.

[29] Kopan R, Ilagan MX (2009). The canonical Notch signaling pathway: unfolding the activation
mechanism. Cell.

[30] Hoey T, Yen WC, Axelrod F, Basi J, Donigian L, Dylla S, Fitch-Bruhns M, Lazetic S, Park IK, Sato A, Satyal S, Wang X, Clarke MF, Lewicki J, Gurney A (2009). DLL4 blockade inhibits tumor growth and reduces tumor-initiating cell
frequency. Cell Stem Cell.

[31] Li K, Li Y, Wu W, Gordon WR, Chang DW, Lu M, Scoggin S, Fu T, Vien L, Histen G, Zheng J, Martin-Hollister R, Duensing T, Singh S, Blacklow SC, Yao Z (2008). Modulation of Notch signaling by antibodies specific for the
extracellular negative regulatory region of NOTCH3. J Biol Chem.

[32] Fan X, Khaki L, Zhu TS, Soules ME, Talsma CE, Gul N, Koh C, Zhang J, Li YM, Maciaczyk J, Nikkhah G, Dimeco F, Piccirillo S, Vescovi AL, Eberhart CG (2010). NOTCH pathway blockade depletes CD133-positive glioblastoma cells and
inhibits growth of tumor neurospheres and xenografts. Stem Cells.

[33] Farnie G, Clarke RB, Spence K, Pinnock N, Brennan K, Anderson NG, Bundred NJ (2007). Novel cell culture technique for primary ductal carcinoma in situ:
role of Notch and epidermal growth factor receptor signaling
pathways. J Natl Cancer Inst.

[34] Wang J, Wakeman TP, Lathia JD, Hjelmeland AB, Wang XF, White RR, Rich JN, Sullenger BA (2010). Notch promotes radioresistance of glioma stem cells. Stem Cells.

[35] Wei P, Walls M, Qiu M, Ding R, Denlinger RH, Wong A, Tsaparikos K, Jani JP, Hosea N, Sands M, Randolph S, Smeal T (2010). Evaluation of selective gamma-secretase inhibitor PF-03084014 for its
antitumor efficacy and gastrointestinal safety to guide optimal clinical trial
design. Mol Cancer Ther.

[36] Ingham PW, McMahon AP (2001). Hedgehog signaling in animal development: paradigms and
principles. Genes Dev.

[37] Varjosalo M, Taipale J (2008). Hedgehog: functions and mechanisms. Genes Dev.

[38] Dierks C, Beigi R, Guo GR, Zirlik K, Stegert MR, Manley P, Trussell C, Schmitt-Graeff A, Landwerlin K, Veelken H, Warmuth M (2008). Expansion of Bcr-Abl-positive leukemic stem cells is dependent on
Hedgehog pathway activation. Cancer Cell.

[39] Hofmann I, Stover EH, Cullen DE, Mao J, Morgan KJ, Lee BH, Kharas MG, Miller PG, Cornejo MG, Okabe R, Armstrong SA, Ghilardi N, Gould S, de Sauvage FJ, McMahon AP, Gilliland DG (2009). Hedgehog signaling is dispensable for adult murine hematopoietic stem
cell function and hematopoiesis. Cell Stem Cell.

[40] Nolan-Stevaux O, Lau J, Truitt ML, Chu GC, Hebrok M, Fernandez-Zapico ME, Hanahan D (2009). GLI1 is regulated through Smoothened-independent mechanisms in
neoplastic pancreatic ducts and mediates PDAC cell survival and
transformation. Genes Dev.

[41] Watkins DN, Berman DM, Burkholder SG, Wang B, Beachy PA, Baylin SB (2003). Hedgehog signalling within airway epithelial progenitors and in
small-cell lung cancer. Nature.

[42] Xie J, Johnson RL, Zhang X, Bare JW, Waldman FM, Cogen PH, Menon AG, Warren RS, Chen LC, Scott MP, Epstein EH (1997). Mutations of the PATCHED gene in several types of sporadic
extracutaneous tumors. Cancer Res.

[43] Xie J, Murone M, Luoh SM, Ryan A, Gu Q, Zhang C, Bonifas JM, Lam CW, Hynes M, Goddard A, Rosenthal A, Epstein EH, de Sauvage FJ (1998). Activating Smoothened mutations in sporadic basal-cell
carcinoma. Nature.

[44] Yauch RL, Gould SE, Scales SJ, Tang T, Tian H, Ahn CP, Marshall D, Fu L, Januario T, Kallop D, Nannini-Pepe M, Kotkow K, Marsters JC, Rubin LL, de Sauvage FJ (2008). A paracrine requirement for hedgehog signalling in
cancer. Nature.

[45] Merchant AA, Matsui W (2010). Targeting Hedgehog--a cancer stem cell pathway. Clin Cancer Res.

[46] Berman DM, Karhadkar SS, Hallahan AR, Pritchard JI, Eberhart CG, Watkins DN, Chen JK, Cooper MK, Taipale J, Olson JM, Beachy PA (2002). Medulloblastoma growth inhibition by hedgehog pathway
blockade. Science.

[47] Han YG, Kim HJ, Dlugosz AA, Ellison DW, Gilbertson RJ, Alvarez-Buylla A (2009). Dual and opposing roles of primary cilia in medulloblastoma
development. Nat Med.

[48] Von Hoff DD, LoRusso PM, Rudin CM, Reddy JC, Yauch RL, Tibes R, Weiss GJ, Borad MJ, Hann CL, Brahmer JR, Mackey HM, Lum BL, Darbonne WC, Marsters JC, de Sauvage FJ, Low JA (2009). Inhibition of the hedgehog pathway in advanced basal-cell
carcinoma. N Engl J Med.

[49] Bailey JM, Mohr AM, Hollingsworth MA (2009). Sonic hedgehog paracrine signaling regulates metastasis and
lymphangiogenesis in pancreatic cancer. Oncogene.

[50] Bhattacharya R, Kwon J, Ali B, Wang E, Patra S, Shridhar V, Mukherjee P (2008). Role of hedgehog signaling in ovarian cancer. Clin Cancer Res.

[51] Fiaschi M, Rozell B, Bergstrom A, Toftgard R (2009). Development of mammary tumors by conditional expression of
GLI1. Cancer Res.

[52] Karhadkar SS, Bova GS, Abdallah N, Dhara S, Gardner D, Maitra A, Isaacs JT, Berman DM, Beachy PA (2004). Hedgehog signalling in prostate regeneration, neoplasia and
metastasis. Nature.

[53] Olive KP, Jacobetz MA, Davidson CJ, Gopinathan A, McIntyre D, Honess D, Madhu B, Goldgraben MA, Caldwell ME, Allard D, Frese KK, Denicola G, Feig C, Combs C, Winter SP, Ireland-Zecchini H (2009). Inhibition of Hedgehog signaling enhances delivery of chemotherapy in
a mouse model of pancreatic cancer. Science.

[54] Tremblay MR, Nevalainen M, Nair SJ, Porter JR, Castro AC, Behnke ML, Yu LC, Hagel M, White K, Faia K, Grenier L, Campbell MJ, Cushing J, Woodward CN, Hoyt J, Foley MA (2008). Semisynthetic cyclopamine analogues as potent and orally bioavailable
hedgehog pathway antagonists. J Med Chem.

[55] Hyman JM, Firestone AJ, Heine VM, Zhao Y, Ocasio CA, Han K, Sun M, Rack PG, Sinha S, Wu JJ, Solow-Cordero DE, Jiang J, Rowitch DH, Chen JK (2009). Small-molecule inhibitors reveal multiple strategies for Hedgehog
pathway blockade. Proc Natl Acad Sci U S A.

[56] Lauth M, Bergstrom A, Shimokawa T, Toftgard R (2007). Inhibition of GLI-mediated transcription and tumor cell growth by
small-molecule antagonists. Proc Natl Acad Sci U S A.

[57] Maun HR, Wen X, Lingel A, de Sauvage FJ, Lazarus RA, Scales SJ, Hymowitz SG (2010). The hedgehog pathway antagonist 5E1 binds hedgehog at the
pseudo-active site. J Biol Chem.

[58] Rijsewijk F, Schuermann M, Wagenaar E, Parren P, Weigel D, Nusse R (1987). The Drosophila homolog of the mouse mammary oncogene int-1 is
identical to the segment polarity gene wingless. Cell.

[59] Cadigan KM, Nusse R (1997). Wnt signaling: a common theme in animal development. Genes Dev.

[60] Clevers H (2006). Wnt/beta-catenin signaling in development and disease. Cell.

[61] Nusse R, Varmus HE (1992). Wnt genes. Cell.

[62] van de Wetering M, Sancho E, Verweij C, de Lau W, Oving I, Hurlstone A, van der Horn K, Batlle E, Coudreuse D, Haramis AP, Tjon-Pon-Fong M, Moerer P, van den Born M, Soete G, Pals S, Eilers M (2002). The beta-catenin/TCF-4 complex imposes a crypt progenitor phenotype on
colorectal cancer cells. Cell.

[63] Willert K, Brown JD, Danenberg E, Duncan AW, Weissman IL, Reya T, Yates JR, Nusse R (2003). Wnt proteins are lipid-modified and can act as stem cell growth
factors. Nature.

[64] van Amerongen R, Mikels A, Nusse R (2008). Alternative wnt signaling is initiated by distinct
receptors. Sci Signal.

[65] Veeman MT, Axelrod JD, Moon RT (2003). A second canon. Functions and mechanisms of beta-catenin-independent
Wnt signaling. Dev Cell.

[66] Wang Y (2009). Wnt/Planar cell polarity signaling: a new paradigm for cancer
therapy. Mol Cancer Ther.

[67] Angers S, Moon RT (2009). Proximal events in Wnt signal transduction. Nat Rev Mol Cell Biol.

[68] Cadigan KM, Liu YI (2006). Wnt signaling: complexity at the surface. J Cell Sci.

[69] Gordon MD, Nusse R (2006). Wnt signaling: multiple pathways, multiple receptors, and multiple
transcription factors. J Biol Chem.

[70] Huang H, He X (2008). Wnt/beta-catenin signaling: new (and old) players and new
insights. Curr Opin Cell Biol.

[71] Polakis P (2007). The many ways of Wnt in cancer. Curr Opin Genet Dev.

[72] Rao TP, Kuhl M (2010). An updated overview on Wnt signaling pathways: a prelude for
more. Circ Res.

[73] Garber K (2009). Drugging the Wnt pathway: problems and progress. J Natl Cancer Inst.

[74] Rey JP, Ellies DL (2010). Wnt modulators in the biotech pipeline. Dev Dyn.

[75] Takahashi-Yanaga F, Kahn M (2010). Targeting Wnt signaling: can we safely eradicate cancer stem
cells?. Clin Cancer Res.

[76] MacDonald BT, Tamai K, He X (2009). Wnt/beta-catenin signaling: components, mechanisms, and
diseases. Dev Cell.

[77] He X, Semenov M, Tamai K, Zeng X (2004). LDL receptor-related proteins 5 and 6 in Wnt/beta-catenin signaling:
arrows point the way. Development.

[78] Kimelman D, Xu W (2006). beta-catenin destruction complex: insights and questions from a
structural perspective. Oncogene.

[79] Kawano Y, Kypta R (2003). Secreted antagonists of the Wnt signalling pathway. J Cell Sci.

[80] Bafico A, Gazit A, Pramila T, Finch PW, Yaniv A, Aaronson SA (1999). Interaction of frizzled related protein (FRP) with Wnt ligands and the
frizzled receptor suggests alternative mechanisms for FRP inhibition of Wnt
signaling. J Biol Chem.

[81] Chang JT, Esumi N, Moore K, Li Y, Zhang S, Chew C, Goodman B, Rattner A, Moody S, Stetten G, Campochiaro PA, Zack DJ (1999). Cloning and characterization of a secreted frizzled-related protein
that is expressed by the retinal pigment epithelium. Hum Mol Genet.

[82] Finch PW, He X, Kelley MJ, Uren A, Schaudies RP, Popescu NC, Rudikoff S, Aaronson SA, Varmus HE, Rubin JS (1997). Purification and molecular cloning of a secreted, Frizzled-related
antagonist of Wnt action. Proc Natl Acad Sci U S A.

[83] Melkonyan HS, Chang WC, Shapiro JP, Mahadevappa M, Fitzpatrick PA, Kiefer MC, Tomei LD, Umansky SR (1997). SARPs: a family of secreted apoptosis-related proteins. Proc Natl Acad Sci U S A.

[84] Hsieh JC, Kodjabachian L, Rebbert ML, Rattner A, Smallwood PM, Samos CH, Nusse R, Dawid IB, Nathans J (1999). A new secreted protein that binds to Wnt proteins and inhibits their
activities. Nature.

[85] Piccolo S, Agius E, Leyns L, Bhattacharyya S, Grunz H, Bouwmeester T, De Robertis EM (1999). The head inducer Cerberus is a multifunctional antagonist of Nodal,
BMP and Wnt signals. Nature.

[86] Bafico A, Liu G, Yaniv A, Gazit A, Aaronson SA (2001). Novel mechanism of Wnt signalling inhibition mediated by Dickkopf-1
interaction with LRP6/Arrow. Nat Cell Biol.

[87] Niehrs C, Shen J (2010). Regulation of Lrp6 phosphorylation. Cell Mol Life Sci.

[88] Sakane H, Yamamoto H, Kikuchi A (2010). LRP6 is internalized by Dkk1 to suppress its phosphorylation in the
lipid raft and is recycled for reuse. J Cell Sci.

[89] Semenov MV, Zhang X, He X (2008). DKK1 antagonizes Wnt signaling without promotion of LRP6
internalization and degradation. J Biol Chem.

[90] Dann CE, Hsieh JC, Rattner A, Sharma D, Nathans J, Leahy DJ (2001). Insights into Wnt binding and signalling from the structures of two
Frizzled cysteine-rich domains. Nature.

[91] Mao B, Wu W, Li Y, Hoppe D, Stannek P, Glinka A, Niehrs C (2001). LDL-receptor-related protein 6 is a receptor for Dickkopf
proteins. Nature.

[92] Pinson KI, Brennan J, Monkley S, Avery BJ, Skarnes WC (2000). An LDL-receptor-related protein mediates Wnt signalling in
mice. Nature.

[93] Tamai K, Semenov M, Kato Y, Spokony R, Liu C, Katsuyama Y, Hess F, Saint-Jeannet JP, He X (2000). LDL-receptor-related proteins in Wnt signal
transduction. Nature.

[94] Witte F, Bernatik O, Kirchner K, Masek J, Mahl A, Krejci P, Mundlos S, Schambony A, Bryja V, Stricker S (2010). Negative regulation of Wnt signaling mediated by CK1-phosphorylated
Dishevelled via Ror2. Faseb J.

[95] Bilic J, Huang YL, Davidson G, Zimmermann T, Cruciat CM, Bienz M, Niehrs C (2007). Wnt induces LRP6 signalosomes and promotes dishevelled-dependent LRP6
phosphorylation. Science.

[96] Davidson G, Wu W, Shen J, Bilic J, Fenger U, Stannek P, Glinka A, Niehrs C (2005). Casein kinase 1 gamma couples Wnt receptor activation to cytoplasmic
signal transduction. Nature.

[97] You L, He B, Xu Z, Uematsu K, Mazieres J, Mikami I, Reguart N, Moody TW, Kitajewski J, McCormick F, Jablons DM (2004). Inhibition of Wnt-2-mediated signaling induces programmed cell death
in non-small-cell lung cancer cells. Oncogene.

[98] Groden J, Thliveris A, Samowitz W, Carlson M, Gelbert L, Albertsen H, Joslyn G, Stevens J, Spirio L, Robertson M (1991). Identification and characterization of the familial adenomatous
polyposis coli gene. Cell.

[99] Kinzler KW, Nilbert MC, Su LK, Vogelstein B, Bryan TM, Levy DB, Smith KJ, Preisinger AC, Hedge P, McKechnie D (1991). Identification of FAP locus genes from chromosome 5q21. Science.

[100] Morin PJ, Sparks AB, Korinek V, Barker N, Clevers H, Vogelstein B, Kinzler KW (1997). Activation of beta-catenin-Tcf signaling in colon cancer by mutations
in beta-catenin or APC. Science.

[101] Korinek V, Barker N, Morin PJ, van Wichen D, de Weger R, Kinzler KW, Vogelstein B, Clevers H (1997). Constitutive transcriptional activation by a beta-catenin-Tcf complex
in APC-/- colon carcinoma. Science.

[102] Luchtenborg M, Weijenberg MP, Wark PA, Saritas AM, Roemen GM, van Muijen GN, de Bruine AP, van den Brandt PA, de Goeij AF (2005). Mutations in APC, CTNNB1 and K-ras genes and expression of hMLH1 in
sporadic colorectal carcinomas from the Netherlands Cohort Study. BMC Cancer.

[103] Eaves CJ, Humphries RK (2010). Acute myeloid leukemia and the Wnt pathway. N Engl J Med.

[104] Nusse R, Fuerer C, Ching W, Harnish K, Logan C, Zeng A, ten Berge D, Kalani Y (2008). Wnt signaling and stem cell control. Cold Spring Harb Symp Quant Biol.

[105] Reya T, Clevers H (2005). Wnt signalling in stem cells and cancer. Nature.

[106] Vermeulen L, De Sousa EMF, van der Heijden M, Cameron K, de Jong JH, Borovski T, Tuynman JB, Todaro M, Merz C, Rodermond H, Sprick MR, Kemper K, Richel DJ, Stassi G, Medema JP (2010). Wnt activity defines colon cancer stem cells and is regulated by the
microenvironment. Nat Cell Biol.

[107] Austin TW, Solar GP, Ziegler FC, Liem L, Matthews W (1997). A role for the Wnt gene family in hematopoiesis: expansion of
multilineage progenitor cells. Blood.

[108] Rattis FM, Voermans C, Reya T (2004). Wnt signaling in the stem cell niche. Curr Opin Hematol.

[109] Reya T, Duncan AW, Ailles L, Domen J, Scherer DC, Willert K, Hintz L, Nusse R, Weissman IL (2003). A role for Wnt signalling in self-renewal of haematopoietic stem
cells. Nature.

[110] Akiri G, Cherian MM, Vijayakumar S, Liu G, Bafico A, Aaronson SA (2009). Wnt pathway aberrations including autocrine Wnt activation occur at
high frequency in human non-small-cell lung carcinoma. Oncogene.

[111] Bafico A, Liu G, Goldin L, Harris V, Aaronson SA (2004). An autocrine mechanism for constitutive Wnt pathway activation in
human cancer cells. Cancer Cell.

[112] Suzuki H, Watkins DN, Jair KW, Schuebel KE, Markowitz SD, Chen WD, Pretlow TP, Yang B, Akiyama Y, Van Engeland M, Toyota M, Tokino T, Hinoda Y, Imai K, Herman JG, Baylin SB (2004). Epigenetic inactivation of SFRP genes allows constitutive WNT
signaling in colorectal cancer. Nat Genet.

[113] Yang ZQ, Liu G, Bollig-Fischer A, Haddad R, Tarca AL, Ethier SP (2009). Methylation-associated silencing of SFRP1 with an 8p11-12
amplification inhibits canonical and non-canonical WNT pathways in breast
cancers. Int J Cancer.

[114] He B, Reguart N, You L, Mazieres J, Xu Z, Lee AY, Mikami I, McCormick F, Jablons DM (2005). Blockade of Wnt-1 signaling induces apoptosis in human colorectal
cancer cells containing downstream mutations. Oncogene.

[115] He B, You L, Uematsu K, Xu Z, Lee AY, Matsangou M, McCormick F, Jablons DM (2004). A monoclonal antibody against Wnt-1 induces apoptosis in human cancer
cells. Neoplasia.

[116] Mikami I, You L, He B, Xu Z, Batra S, Lee AY, Mazieres J, Reguart N, Uematsu K, Koizumi K, Jablons DM (2005). Efficacy of Wnt-1 monoclonal antibody in sarcoma cells. BMC Cancer.

[117] You L, He B, Uematsu K, Xu Z, Mazieres J, Lee A, McCormick F, Jablons DM (2004). Inhibition of Wnt-1 signaling induces apoptosis in
beta-catenin-deficient mesothelioma cells. Cancer Res.

[118] You L, He B, Xu Z, Uematsu K, Mazieres J, Fujii N, Mikami I, Reguart N, McIntosh JK, Kashani-Sabet M, McCormick F, Jablons DM (2004). An anti-Wnt-2 monoclonal antibody induces apoptosis in malignant
melanoma cells and inhibits tumor growth. Cancer Res.

[119] DeAlmeida VI, Miao L, Ernst JA, Koeppen H, Polakis P, Rubinfeld B (2007). The soluble wnt receptor Frizzled8CRD-hFc inhibits the growth of
teratocarcinomas in vivo. Cancer Res.

[120] Mao B, Wu W, Davidson G, Marhold J, Li M, Mechler BM, Delius H, Hoppe D, Stannek P, Walter C, Glinka A, Niehrs C (2002). Kremen proteins are Dickkopf receptors that regulate Wnt/beta-catenin
signalling. Nature.

[121] Bourhis E, Tam C, Franke Y, Bazan JF, Ernst J, Hwang J, Costa M, Cochran AG, Hannoush RN (2010). Reconstitution of a frizzled8.Wnt3a.LRP6 signaling complex reveals
multiple Wnt and Dkk1 binding sites on LRP6. J Biol Chem.

[122] Zeng X, Huang H, Tamai K, Zhang X, Harada Y, Yokota C, Almeida K, Wang J, Doble B, Woodgett J, Wynshaw-Boris A, Hsieh JC, He X (2008). Initiation of Wnt signaling: control of Wnt coreceptor Lrp6
phosphorylation/activation via frizzled, dishevelled and axin
functions. Development.

[123] Grandy D, Shan J, Zhang X, Rao S, Akunuru S, Li H, Zhang Y, Alpatov I, Zhang XA, Lang RA, Shi DL, Zheng JJ (2009). Discovery and characterization of a small molecule inhibitor of the
PDZ domain of dishevelled. J Biol Chem.

[124] Fujii N, You L, Xu Z, Uematsu K, Shan J, He B, Mikami I, Edmondson LR, Neale G, Zheng J, Guy RK, Jablons DM (2007). An antagonist of dishevelled protein-protein interaction suppresses
beta-catenin-dependent tumor cell growth. Cancer Res.

[125] Hino S, Michiue T, Asashima M, Kikuchi A (2003). Casein kinase I epsilon enhances the binding of Dvl-1 to Frat-1 and is
essential for Wnt-3a-induced accumulation of beta-catenin. J Biol Chem.

[126] Liu W, Dong X, Mai M, Seelan RS, Taniguchi K, Krishnadath KK, Halling KC, Cunningham JM, Boardman LA, Qian C, Christensen E, Schmidt SS, Roche PC, Smith DI, Thibodeau SN (2000). Mutations in AXIN2 cause colorectal cancer with defective mismatch
repair by activating beta-catenin/TCF signalling. Nat Genet.

[127] Oates NA, van Vliet J, Duffy DL, Kroes HY, Martin NG, Boomsma DI, Campbell M, Coulthard MG, Whitelaw E, Chong S (2006). Increased DNA methylation at the AXIN1 gene in a monozygotic twin from
a pair discordant for a caudal duplication anomaly. Am J Hum Genet.

[128] Satoh S, Daigo Y, Furukawa Y, Kato T, Miwa N, Nishiwaki T, Kawasoe T, Ishiguro H, Fujita M, Tokino T, Sasaki Y, Imaoka S, Murata M, Shimano T, Yamaoka Y, Nakamura Y (2000). AXIN1 mutations in hepatocellular carcinomas, and growth suppression
in cancer cells by virus-mediated transfer of AXIN1. Nat Genet.

[129] Huang SM, Mishina YM, Liu S, Cheung A, Stegmeier F, Michaud GA, Charlat O, Wiellette E, Zhang Y, Wiessner S, Hild M, Shi X, Wilson CJ, Mickanin C, Myer V, Fazal A (2009). Tankyrase inhibition stabilizes axin and antagonizes Wnt
signalling. Nature.

[130] Hsiao SJ, Smith S (2008). Tankyrase function at telomeres, spindle poles, and
beyond. Biochimie.

[131] Chen B, Dodge ME, Tang W, Lu J, Ma Z, Fan CW, Wei S, Hao W, Kilgore J, Williams NS, Roth MG, Amatruda JF, Chen C, Lum L (2009). Small molecule-mediated disruption of Wnt-dependent signaling in
tissue regeneration and cancer. Nat Chem Biol.

[132] Schreinemachers DM, Everson RB (1994). Aspirin use and lung, colon, and breast cancer incidence in a
prospective study. Epidemiology.

[133] Thun MJ, Namboodiri MM, Heath CW (1991). Aspirin use and reduced risk of fatal colon cancer. N Engl J Med.

[134] Thun MJ, Henley SJ, Patrono C (2002). Nonsteroidal anti-inflammatory drugs as anticancer agents:
mechanistic, pharmacologic, and clinical issues. J Natl Cancer Inst.

[135] Grosch S, Tegeder I, Niederberger E, Brautigam L, Geisslinger G (2001). COX-2 independent induction of cell cycle arrest and apoptosis in
colon cancer cells by the selective COX-2 inhibitor celecoxib. Faseb J.

[136] Jaiswal AS, Marlow BP, Gupta N, Narayan S (2002). Beta-catenin-mediated transactivation and cell-cell adhesion pathways
are important in curcumin (diferuylmethane)-induced growth arrest and apoptosis in
colon cancer cells. Oncogene.

[137] Kim J, Zhang X, Rieger-Christ KM, Summerhayes IC, Wazer DE, Paulson KE, Yee AS (2006). Suppression of Wnt signaling by the green tea compound
(-)-epigallocatechin 3-gallate (EGCG) in invasive breast cancer cells. Requirement
of the transcriptional repressor HBP1. J Biol Chem.

[138] Abrami L, Kunz B, Iacovache I, van der Goot FG (2008). Palmitoylation and ubiquitination regulate exit of the Wnt signaling
protein LRP6 from the endoplasmic reticulum. Proc Natl Acad Sci U S A.

[139] Chamoun Z, Mann RK, Nellen D, von Kessler DP, Bellotto M, Beachy PA, Basler K (2001). Skinny hedgehog, an acyltransferase required for palmitoylation and
activity of the hedgehog signal. Science.

[140] Kurayoshi M, Yamamoto H, Izumi S, Kikuchi A (2007). Post-translational palmitoylation and glycosylation of Wnt-5a are
necessary for its signalling. Biochem J.

[141] Takada R, Satomi Y, Kurata T, Ueno N, Norioka S, Kondoh H, Takao T, Takada S (2006). Monounsaturated fatty acid modification of Wnt protein: its role in
Wnt secretion. Dev Cell.

[142] Daniels DL, Weis WI (2005). Beta-catenin directly displaces Groucho/TLE repressors from Tcf/Lef in
Wnt-mediated transcription activation. Nat Struct Mol Biol.

[143] Mosimann C, Hausmann G, Basler K (2009). Beta-catenin hits chromatin: regulation of Wnt target gene
activation. Nat Rev Mol Cell Biol.

[144] Willert K, Jones KA (2006). Wnt signaling: is the party in the nucleus?. Genes Dev.

[145] Eguchi M, Nguyen C, Lee SC, Kahn M (2005). ICG-001, a novel small molecule regulator of TCF/beta-catenin
transcription. Med Chem.

[146] Emami KH, Nguyen C, Ma H, Kim DH, Jeong KW, Eguchi M, Moon RT, Teo JL, Kim HY, Moon SH, Ha JR, Kahn M (2004). A small molecule inhibitor of beta-catenin/CREB-binding protein
transcription [corrected]. Proc Natl Acad Sci U S A.

[147] Henderson WR, Chi EY, Ye X, Nguyen C, Tien YT, Zhou B, Borok Z, Knight DA, Kahn M (2010). Inhibition of Wnt/{beta}-catenin/CREB binding protein (CBP) signaling
reverses pulmonary fibrosis. Proc Natl Acad Sci U S A.

[148] Lepourcelet M, Chen YN, France DS, Wang H, Crews P, Petersen F, Bruseo C, Wood AW, Shivdasani RA (2004). Small-molecule antagonists of the oncogenic Tcf/beta-catenin protein
complex. Cancer Cell.

[149] Mahmoudi T, Li VS, Ng SS, Taouatas N, Vries RG, Mohammed S, Heck AJ, Clevers H (2009). The kinase TNIK is an essential activator of Wnt target
genes. Embo J.

[150] Shitashige M, Satow R, Jigami T, Aoki K, Honda K, Shibata T, Ono M, Hirohashi S, Yamada T (2010). Traf2- and Nck-interacting kinase is essential for Wnt signaling and
colorectal cancer growth. Cancer Res.

[151] Malanchi I, Peinado H, Kassen D, Hussenet T, Metzger D, Chambon P, Huber M, Hohl D, Cano A, Birchmeier W, Huelsken J (2008). Cutaneous cancer stem cell maintenance is dependent on beta-catenin
signalling. Nature.

[152] Watt FM, Collins CA (2008). Role of beta-catenin in epidermal stem cell expansion, lineage
selection, and cancer. Cold Spring Harb Symp Quant Biol.

[153] Zeng YA, Nusse R (2010). Wnt proteins are self-renewal factors for mammary stem cells and
promote their long-term expansion in culture. Cell Stem Cell.

[154] Balic M, Lin H, Young L, Hawes D, Giuliano A, McNamara G, Datar RH, Cote RJ (2006). Most early disseminated cancer cells detected in bone marrow of breast
cancer patients have a putative breast cancer stem cell phenotype. Clin Cancer Res.

[155] Brabletz T, Jung A, Spaderna S, Hlubek F, Kirchner T (2005). Opinion: migrating cancer stem cells - an integrated concept of
malignant tumour progression. Nat Rev Cancer.

[156] Dean M, Fojo T, Bates S (2005). Tumour stem cells and drug resistance. Nat Rev Cancer.

[157] Frank NY, Margaryan A, Huang Y, Schatton T, Waaga-Gasser AM, Gasser M, Sayegh MH, Sadee W, Frank MH (2005). ABCB5-mediated doxorubicin transport and chemoresistance in human
malignant melanoma. Cancer Res.

[158] Hermann PC, Huber SL, Herrler T, Aicher A, Ellwart JW, Guba M, Bruns CJ, Heeschen C (2007). Distinct populations of cancer stem cells determine tumor growth and
metastatic activity in human pancreatic cancer. Cell Stem Cell.

[159] Dylla SJ, Beviglia L, Park IK, Chartier C, Raval J, Ngan L, Pickell K, Aguilar J, Lazetic S, Smith-Berdan S, Clarke MF, Hoey T, Lewicki J, Gurney AL (2008). Colorectal cancer stem cells are enriched in xenogeneic tumors
following chemotherapy. PLoS One.

[160] Li X, Lewis MT, Huang J, Gutierrez C, Osborne CK, Wu MF, Hilsenbeck SG, Pavlick A, Zhang X, Chamness GC, Wong H, Rosen J, Chang JC (2008). Intrinsic resistance of tumorigenic breast cancer cells to
chemotherapy. J Natl Cancer Inst.

[161] Al-Hajj M, Wicha MS, Benito-Hernandez A, Morrison SJ, Clarke MF (2003). Prospective identification of tumorigenic breast cancer
cells. Proc Natl Acad Sci U S A.

[162] Liebner S, Cattelino A, Gallini R, Rudini N, Iurlaro M, Piccolo S, Dejana E (2004). Beta-catenin is required for endothelial-mesenchymal transformation
during heart cushion development in the mouse. J Cell Biol.

[163] Kim K, Lu Z, Hay ED (2002). Direct evidence for a role of beta-catenin/LEF-1 signaling pathway in
induction of EMT. Cell Biol Int.

[164] Muller T, Bain G, Wang X, Papkoff J (2002). Regulation of epithelial cell migration and tumor formation by
beta-catenin signaling. Exp Cell Res.

[165] Thiery JP, Acloque H, Huang RY, Nieto MA (2009). Epithelial-mesenchymal transitions in development and
disease. Cell.

[166] Mani SA, Guo W, Liao MJ, Eaton EN, Ayyanan A, Zhou AY, Brooks M, Reinhard F, Zhang CC, Shipitsin M, Campbell LL, Polyak K, Brisken C, Yang J, Weinberg RA (2008). The epithelial-mesenchymal transition generates cells with properties
of stem cells. Cell.

