# Erratum: Drug Discovery Approaches to Target Wnt Signaling in Cancer Stem Cells

**DOI:** 10.18632/oncotarget.26220

**Published:** 2018-10-05

**Authors:** Joshua C. Curtin, Matthew V. Lorenzi

**Affiliations:** ^1^ Oncology Drug Discovery, Research and Development, Bristol-Myers Squibb, Princeton, NJ, USA

**This article has been corrected:** During production, the page numbers for this article were listed incorrectly. The page numbers have now been adjusted to show the proper pagination.

Original article: Oncotarget. 2010; 1:563-577. https://doi.org/10.18632/oncotarget.191

